# Roles of NAC transcription factors in the regulation of biotic and abiotic stress responses in plants

**DOI:** 10.3389/fmicb.2013.00248

**Published:** 2013-09-03

**Authors:** Mohammed Nuruzzaman, Akhter M. Sharoni, Shoshi Kikuchi

**Affiliations:** ^1^Plant Genome Research Unit, Division of Genome and Biodiversity Research, Agrogenomics Research Center, National Institute of Agrobiological SciencesTsukuba, Japan; ^2^Graduate School of Science and Engineering, Institute for Environmental Science and Technology, Saitama UniversitySaitama, Japan

**Keywords:** phylogenetic analysis, motif, NAC transcription factors, defense signaling pathways, biotic infections, abiotic stresses

## Abstract

NAC transcription factors are one of the largest families of transcriptional regulators in plants, and members of the *NAC* gene family have been suggested to play important roles in the regulation of the transcriptional reprogramming associated with plant stress responses. A phylogenetic analysis of *NAC* genes, with a focus on rice and Arabidopsis, was performed. Herein, we present an overview of the regulation of the stress responsive *NAC SNAC*/(*IX*) group of genes that are implicated in the resistance to different stresses. SNAC factors have important roles for the control of biotic and abiotic stresses tolerance and that their overexpression can improve stress tolerance via biotechnological approaches. We also review the recent progress in elucidating the roles of NAC transcription factors in plant biotic and abiotic stresses. Modification of the expression pattern of transcription factor genes and/or changes in their activity contribute to the elaboration of various signaling pathways and regulatory networks. However, a single *NAC* gene often responds to several stress factors, and their protein products may participate in the regulation of several seemingly disparate processes as negative or positive regulators. Additionally, the NAC proteins function via auto-regulation or cross-regulation is extensively found among *NAC* genes. These observations assist in the understanding of the complex mechanisms of signaling and transcriptional reprogramming controlled by NAC proteins.

## Introduction

Biotic and abiotic stresses trigger a wide range of plant responses, from the alteration of gene expression and cellular metabolism to changes in plant growth and development and crop yields. Transcription factors (TFs) and *cis*-elements function in the promoter region of different stress-related genes, and the overexpression or suppression of these genes may improve the plant's tolerance to both types of stress. The NAC acronym is derived from three genes that were initially discovered to contain a particular domain (the NAC domain): NAM (for no apical meristem), ATAF1 and −2, and CUC2 (for cup-shaped cotyledon) (Souer et al., 1996; Aida et al., 1997). The *NAC* genes constitute one of the largest families of plant-specific TFs and are present in a wide range of species. Extensive investigation aided by the availability of several complete plant genomic sequences has identified 117 *NAC* genes in Arabidopsis, 151 in rice, 79 in grape, 26 in citrus, 163 in poplar, and 152 each in soybean and tobacco(Rushton et al., 2008; Hu et al., 2010; Nuruzzaman et al., 2010, 2012a; Le et al., 2011).

In the past decade, significant progress has been achieved in determining the molecular mechanisms of innate immune responses in rice, host recognition of pathogens, recognition-triggered early signaling events, and signaling pathways and their involvement in activating defense responses (Skamnioti and Gurr, 2009; Liu et al., 2010; Valent and Khang, 2010). To date, numerous studies have elucidated the regulatory mechanism of innate immune response in rice against blast disease, which is caused by *Magnaporthe* (M) *oryzae*. Multiple disease resistance genes (*R* genes) have been cloned and characterized (Liu et al., 2010). Similar to Arabidopsis, the salicylic acid (SA) and ethylene (ET)/jasmonic acid (JA)-mediated signaling pathways are critical in activating innate immune responses in rice and can operate in concert using some common components or biochemical events (Chern et al., 2005; Qiu et al., 2007; Yuan et al., 2007; Li et al., 2011). A number of regulatory proteins, including several TFs (e.g., OsNAC6), function in regulating defense responses against *M. grisea* (Nakashima et al., 2007). However, a complete understanding of the molecular network regulating the rice immune responses against pathogens remains unclear. Microarray profiling after biotic treatments [rice stripe virus (RSV) and rice tungro spherical virus (RTSV)] in rice seedlings has revealed six *OsNAC* genes induced by both virus infections (Nuruzzaman et al., 2010). Rice plants with a mutation in *rim1-1* are resistant to infection by dwarf virus (Yoshii et al., 2009; Saga et al., 2011). The *StNAC* (*Solanum tuberosum*) gene is induced in response to *Phytophthora infestans* infection (Collinge and Boller, 2001). Furthermore, numerous *NAC* genes are involved in the response of plants to abiotic stresses, such as drought, salinity, cold, and submergence (Hu et al., 2006; Jeong et al., 2010; Nuruzzaman et al., 2012b).

Genes in the NAC family have been shown to regulate a wide range of developmental processes, including seed development (Sperotto et al., 2009), embryo development (Duval et al., 2002), shoot apical meristem formation (Kim et al., 2007a), fiber development (Ko et al., 2007), leaf senescence (Guo et al., 2005; Breeze et al., 2011), and cell division (Kim et al., 2006). Additionally, expression of the *AtNAC1* gene is induced by lateral root development, which in turn is regulated by the hormone auxin (Xie et al., 2000).

Regardless, few of these genes have been characterized to date. Indeed, most of the NAC family members have yet to be characterized, even though these genes are likely to play important roles in plant physiology, and substantial experimental work will be required to determine the specific biological function of each *NAC* gene. Based on phylogenetic analyses, it is apparent that this large family of TFs consists of groups that are closely related to each other (Kranz et al., 1998; Reyes et al., 2004; Tian et al., 2004). The focus of this review is the phylogeny of *NAC* genes with respect to resistance pathways. We also present an overview of the regulation of the *SNAC*/(*IX*) group of genes that are implicated in the resistance to different stresses. Furthermore, we will emphasize on the roles of *NAC* TFs genes in plant biotic and abiotic stresses.

## Structural features of the NAC proteins

The N-terminus of NAC proteins is a highly homologous region containing the DNA-binding NAC domain. NAC proteins commonly possess a conserved NAC domain at the N-terminus that consists of approximately 150–160 amino acids and is divided into five sub-domains (A to E) (Ooka et al., 2003). The function of the NAC domain has been associated with nuclear localization, DNA binding, and the formation of homodimers or heterodimers with other NAC domain-containing proteins (Olsen et al., 2005). The structure of the DNA-binding NAC domain of Arabidopsis *ANAC019* has been solved by X-ray crystallography (Ernst et al., 2004), and the functional dimer formed by the NAC domain was identified in the structural analysis. The NAC domain structure of a rice stress-responsive NAC protein (SNAC1; STRESS-RESPONSIVE NAC 1) was also reported (Chen et al., 2011) and shares structural similarity with the NAC domain from Arabidopsis *ANAC019*. In contrast, the C-terminal regions of NAC proteins are highly divergent (Ooka et al., 2003) and are responsible for the observed regulatory differences between the transcriptional activation activity of NAC proteins (Xie et al., 2000; Yamaguchi et al., 2008; Jensen et al., 2010). The divergent C-terminal region of these proteins generally operates as a functional domain, acting as a transcriptional activator or repressor (Tran et al., 2004; Hu et al., 2006; Kim et al., 2007b). The C-terminal region is large and possesses protein-binding activity.

## Structural conservation of SNAC group

**Figure 1:**
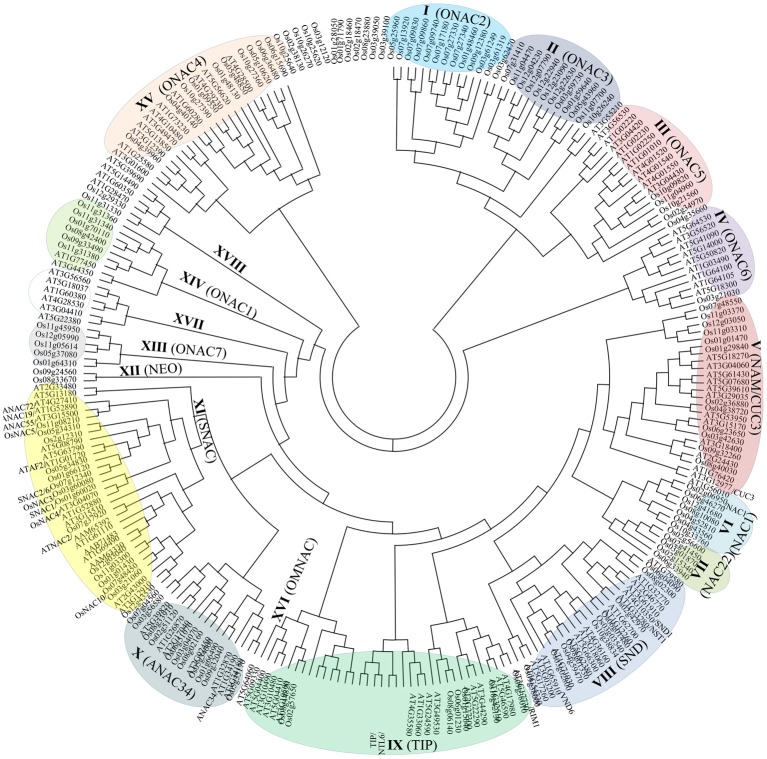
**An unrooted phylogenetic tree of the NAC transcription factors of rice and Arabidopsis.** The amino acid sequences of the NAC domain of 135 rice NAC family proteins and 117 Arabidopsis NAC proteins were aligned by ClustalW, and the phylogenetic tree was constructed using MEGA 4.0 and the NJ method. Bootstrap values from 1000 replicates were used to assess the robustness of the trees. The classification by Nuruzzaman et al. (2010) is indicated in parentheses.

**Figure 2:**
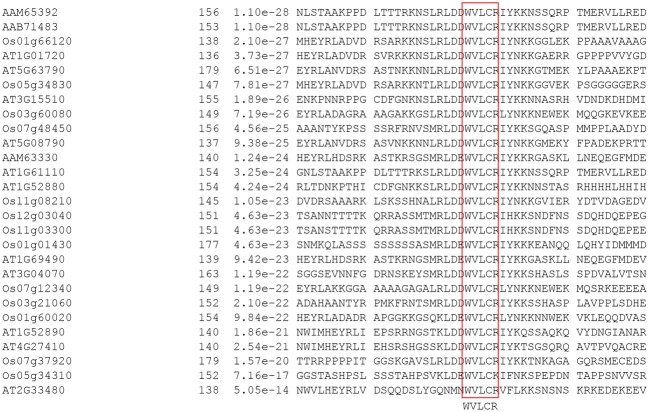
**Conserved motifs outside of the NAC domain of the SNAC/(IX) group in rice and Arabidopsis**.

The evolutionary analysis of developmental processes of *NAC* genes through the correlation of function and phylogeny is a well-known approach in plant research (Figure 1; Nuruzzaman et al., 2010, 2012a). The NAC TF family has experienced extensive expansion through gene duplication events. Although NAC structural diversity has been constrained within the 60-amino acid conserved domain, which comprises a unique DNA-interacting β-sheet structure, structural conservation outside this conserved domain is extremely limited. Additional highly conserved motifs can be identified only within specific groups (e.g., SNAC, TIP, and SND), and most members in the same group share one or more motifs outside the NAC domain (Nuruzzaman et al., 2012a). A phylogeny of the SNAC group, which includes the *ANAC019* and *OsNAC6* genes, indicates the existence of multiple co-orthologs in dicots and monocots (Figure 1). Indeed, the SNAC group has some highly conserved motifs (Figure 2) within regions outside the conserved domain. A 28-amino acid (WVLCR) motif (RSARKKNSLRLDDWVLCRIYNKKGGLEK in OsNAC) is found amino-terminal to the conserved DNA-binding domain in monocots and in dicots. We first identified putative conserved motifs outside of the NAC domain in rice and compared with those of Arabidopsis and citrus. Outside of the NAC domain, rice specific conserved motifs were detected (Nuruzzaman et al., 2012a). These conserved motifs are likely to be involved in the recruitment of proteins that are involved in activating gene expression or perhaps in the control of protein stability. It is notable that only some of these motifs are conserved in both dicots and monocots, suggesting that protein function has both diverged and been conserved even within this evolutionarily conserved NAC family. Further analysis of motif function via protein-interaction analyses of TF complexes is needed.

## Roles played by NAC transcription factors

Since the early research into NAC TFs, it was evident that these factors play roles in regulating several different plant processes. For convenience, some of these processes are discussed individually below. The recent data presented here provided new insight, namely, that it is common for a single NAC NF to regulate transcriptional reprogramming that is associated with multiple plant programs: the dynamic web of signaling in which NAC factors operate has multiple inputs and outputs.

### NAC function in biotic stress

**Figure 3:**
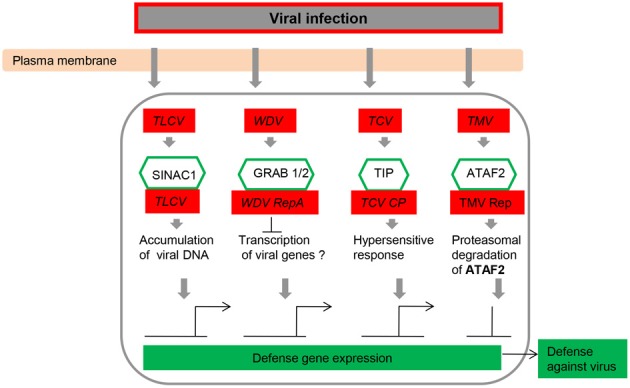
**NAC transcription factors as key components in the transcriptional regulation of gene expression during virus infection.** Abbreviations: TCV, turnip crinkle virus; TIP, TCV-interacting protein; TLCV, tomato leaf curl virus; TMV, tobacco mosaic virus; WDV, wheat dwarf geminivirus.

**Figure 4:**
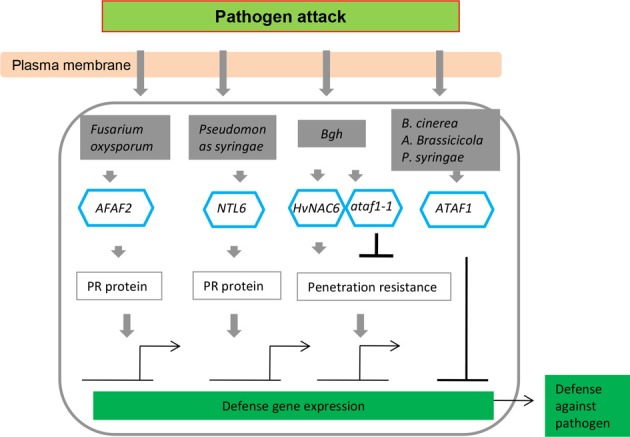
**NAC transcription factors as key components in transcriptional regulation of gene expression during pathogen attack, integrating both positive (arrows) and negative (bars) regulatory mechanisms**.

**Figure 5:**
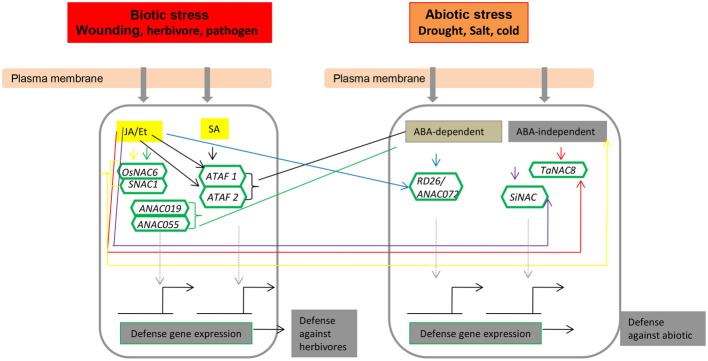
**The role of NAC transcription factors in the herbivore/biotic and abiotic response signaling pathway.** Key to all colors: OsNAC6/SNAC1, yellow; ANAC019/ANAC055, green; ATAF1/ATAF2, black; TaNAC8, red; SiNAC, purple; RD26/ANAC072, blue. Abbreviations: ABA, abscisic acid; ANAC, *Arabidopsis thaliana* NAC; JA, jasmonic acid; Et, ethylene; and SA, salicylic acid.

The majority of reports concerning NAC TFs have indicated that numerous members of the multigene family play roles in the transcriptional reprogramming associated with plant immune responses. This is an active research area that has been extensively reviewed and therefore will only be briefly considered here. To date, it is clear that NAC NFs are central components of many aspects of the plant innate immune system, basal defense, and systemic acquired resistance. There are many examples in which the overexpression or knockdown of *NAC* gene expression has effects on plant defense, observations that have allowed the resolution of some components of the web of signaling pathways (Figures 3–5; Table 1) (Collinge and Boller, 2001; Delessert et al., 2005; He et al., 2005; Jensen et al., 2007, 2008, 2010).

**Table 1:** **Function of NAC transcription factors in biotic infections**.

**Genes/target genes**	**Functions**	**Method**	**Species**	**References**
*HvNAC6*	*HvNAC6* positively regulates penetration resistant toward *Bl. gramini f.sp. hordei (Bgh)* attack	Knockdown/overexpression	*H. vulgare*	Jensen et al., 2007
*ataf1-1*	Loss-of-function mutants have attenuated penetration resistance toward *Bgh* attack	Knockout	*A. thaliana (At)*	Jensen et al., 2008
*ATAF1, PR1*	ATAF1 negatively regulates resistance to *B. cinerea*	Overexpression/*ataf1-1* and *ataf1-2*, knockout	*A. thaliana*	Wu et al., 2009
*ATAF1, PR-1, PR-5, NPR1, PDF1.2*	ATAF1 negatively regulates resistance to *P. syringae*, *B. cinerea*, *A. brassicicola*	Overexpression/*ataf1-2*, knockout	*A. thaliana*	Wang et al., 2009a
*ATAF2, PR1, PR2, PR4, PR5, PDF1.1, PDF1.2*	ATAF2 negatively regulates resistance to *F. oxysporum*, represses pathogenesis-related proteins	Overexpression/knockout	*A. thaliana*	Delessert et al., 2005
*ATAF2, PR1, PR2, PDF1.2*	OX = Reduced tobacco mosaic virus accumulation, increased pathogenesis-related genes	Overexpression/knockout	*A. thaliana*	Wang et al., 2009a
*ATAF2, NIT2*	Defense hormones, pathogen infection	Overexpression/knockout	*A. thaliana*	Huh et al., 2012
*ANAC019, ANAC055*	Defense disease, JA pathway	Overexpression	*A. thaliana*	Bu et al., 2008
*NTL6, PR1, PR2, PR5*	Positive regulator of pathogen resistance against *P. syringae*	Gene silencing/overexpression	*A. thaliana*	Seo et al., 2010
*ANAC042, P450*	Regulation of camalexin biosynthesis, pathogen infection	β - Glucuronidase (GUS)-reporter assays	*A. thaliana*	Saga et al., 2012
*SlNAC1*	Increased tomato leaf curl virus (TLCV) DNA accumulation	Transient overexpression	*N. benthamiana*	Selth et al., 2005
*OsNAC4*	Inducer of HR cell death upon *Acidovorax avenae* infection, loss of plasma membrane integrity, nuclear DNA fragmentation	Overexpression/knockdown	*Oryza (O) sativa*	Kaneda et al., 2009
*OsNAC6, PR protein 1, Probenazoleinducible proteins (PBZ1s), DUF26- like Ser/Thr protein kinase, Thioredoxin, Peroxidase, Lipoxygenase,*	Slightly increased tolerance to rice blast disease	Overexpression	*O. sativa*	Nakashima et al., 2007
*rim1-1*	Resistance to rice dwarf virus (RDV), susceptible to *rice* transitory yellowing virus (RTYV) and RSV	Knockout	*O. sativa*	Yoshii et al., 2009
*Os02g34970, Os02g38130, Os11g03310, Os11g03370, Os11g05614, Os12g03050*	RSV, RTSV infections	Microarray	*O. sativa*	Nuruzzaman et al., 2010
*OsNAC19*	Disease resistance	Infection	*O. sativa*	Lin et al., 2007
*GRAB1, GRAB2*	Inhibited wheat dwarf virus replication	Transient Overexpression	*T. monococcum*	Xie et al., 1999
*ATAF2*	Tobacco mosaic virus	Transgenic	*Tobaco*	Wang et al., 2009b
*ONAC122* and *ONAC131* brome mosaic virus (BMV)	Defense responses against *Magnaporthe grisea*	–	*O. sativa*	Sun et al., 2013
*SlNAC1*	Upregulated during pseudomonas infection	Pathogen infection	*S. lycopersicum*	Huang et al., 2012
*CaNAC1*	Defense responses against pathogen	Infection	*C. arietinum*	Oh et al., 2005
*GmNAC6*	Responses to biotic signals, osmotic stress-induced	Transctiption	*G. max*	Faria et al., 2011
*TLCV, SlNAC1*	Enhances viral replication	Overexpression	*L. esculentum*	Selth et al., 2005
*BnNAC14, BnNAC485, ATAF1 or ATAF2*	Response to biotic and abiotic stresses including wounding	cDNA libraries	*–*	Hegedus et al., 2003
*Stprx2, StNAC*	Wounding and pathogen response	Transcriptome	*S. tuberosum*	Collinge and Boller, 2001
*NT L4*	ROS under abscisic acid, leaf senescence	Transgenic	*A. thaliana*	Lee et al., 2012
*NTL9*	Osmotic stress responses, leaf senescence	Overexpression/knocout	*A. thaliana*	Yoon et al., 2008
*MtNAC969*	Symbiotic nodule senescence	Overexpresion	*M. truncatula*	de Zélicourt et al., 2012
*VNI2, OR/RD*	Leaf senescence	Transcription	*A. thaliana*	Seo and Park, 2011
*Os07g37920, Wheat GPC*	Senescence	Overexpression/RNAi	*O. sativa, T. aestivum*	Distelfeld et al., 2012
*AtNAP*	Leaf senescence	Overexpression/RNAi	*A. thaliana*	Guo and Gan, 2006

### Regulation of NAC TFs by pathogen infection

Sun and co-workers applied Virus-induced gene silencing (VIGS) system to investigate the function of NAC TFs (ONAC122 and ONAC131) in disease resistance against *M. grisea* (Sun et al., 2013). VIGS is a useful tool for the rapid analysis of gene function in plants (Liu et al., 2002; Purkayastha and Dasgupta, 2009; Scofield and Nelson, 2009). Some VIGS vectors have been developed for dicotyledonous plants among which the tobacco rattle virus (TRV)-based VIGS vector is the most successful example for members of Solanaceae, such as *Nicotiana benthamiana* and *Lycopersicon esculentum* (Liu et al., 2002; Chakravarthy et al., 2010). The barley stripe mosaic virus (BSMV)-based VIGS vector was used to characterize multiple genes for their roles in disease resistance in wheat and barley (Hein et al., 2005; Scofield et al., 2005; Zhou et al., 2007; Sindhu et al., 2008). Several scientists have developed a brome mosaic virus (BMV)-based VIGS vector, and this vector was demonstrated to be a versatile tool for rapid gene function analysis in barley, rice, and maize (Ding et al., 2006; Pacak et al., 2010; van der Linde et al., 2011; Biruma et al., 2012). In rice seedlings, 19 and 13 *NAC* genes were up-regulated after RSV and RTSV infection, respectively, at different days after inoculation (Nuruzzaman et al., 2010). Several NAC proteins can either enhance or inhibit virus multiplication by directly interacting with virus-encoded proteins (Figure 3; Xie et al., 1999; Ren et al., 2000, 2005; Selth et al., 2005; Jeong et al., 2008; Yoshii et al., 2009), and increases in the expression level of *NAC* genes have been monitored in response to attack by viruses, several fungal elicitors, and bacteria (Figures 3, 4; Xie et al., 1999; Ren et al., 2000; Collinge and Boller, 2001; Mysore et al., 2002; Hegedus et al., 2003; Oh et al., 2005; Selth et al., 2005; Jensen et al., 2007; Lin et al., 2007; Jeong et al., 2008; Wang et al., 2009a,b; Xia et al., 2010a,b). Such dual modulation in plant defense implies the association of NAC proteins with distinct regulatory complexes.

Kaneda et al. (2009) reported that *OsNAC4* is a key positive regulator of hypersensitive cell death in plants, and hypersensitive cell death is markedly decreased in response to avirulent bacterial strains in *OsNAC4*-knock-down lines. After induction by an avirulent pathogen recognition signal, *OsNAC4* is translocated into the nucleus in a phosphorylation-dependent manner. Conversely, the overexpression of *OsNAC6* does not lead to hypersensitive cell death (Kaneda et al., 2009), whereas transgenic rice plants overexpressing *OsNAC6* exhibited tolerance to blast disease (Nakashima et al., 2007). *ATAF2* overexpression resulted in increased susceptibility toward the necrotrophic fungus *Fusarium oxysporum* under sterile conditions due to the repression of pathogenesis-related (*PR*) genes (Delessert et al., 2005) but induced *PR* genes, reducing tobacco mosaic virus accumulation in a non-sterile environment (Wang et al., 2009b). RNA interference and overexpression studies have also revealed the function of NAC TFs in various plant–pathogen interactions (Figure 4). A number of NAC proteins may positively regulate plant defense responses by activating *PR* genes, inducing a hypersensitive response (HR), and cell death at the infection site (Figure 4; Jensen et al., 2007, 2008; Kaneda et al., 2009; Seo et al., 2010). *ATAF1* and its barley homolog *HvNAC6* positively regulate penetration resistance to the biotrophic fungus *Blumeria graminis* f.sp. *hordei* (*Bgh*) (Jensen et al., 2007, 2008) but attenuate the resistance to other pathogens, such as *Pseudomonas syringae*, *Botrytis cinerea*, and *Alternaria brassicicola* (Wang et al., 2009a; Wu et al., 2009). Unlike *ATAF2*, *ATAF1* and *HvNAC6* are transcriptional activators and may indirectly regulate the repression of *PR* genes via a hypothetical negative regulator (Figure 4). Hence, the ATAF subfamily clearly appears to have a conserved but non-redundant function in regulating the responses to different pathogens. The immune response in plants elicited upon pathogen infection is characterized by activation of multiple defense responses including expression of a large set of defense-related genes (van Loon et al., 2006), which are regulated by different types of TFs. Many TFs belonging to the NAC, ERF, and WRKY families have been identified (Eulgem and Somssich, 2007; Gutterson and Reuber, 2004) and revealed to play important roles in regulating expression of defense-related genes.

Arabidopsis stress-responsive *NAC* genes, such as *RD26*, respond to JA, a well-described phytohormone that is functionally involved in regulating wounding and biotic stress responses (Fujita et al., 2004, 2006). Hence, it is reasonable to consider that JA-responsive SNAC factors might function in both biotic and abiotic stress responses. In rice, most of the genes in the SNAC group respond to JA. Among them, *SNAC1*, *OsNAC3*, *OsNAC4*, *OsNAC5*, *OsNAC6*, and *OsNAC10* are present in the same phylogenetic SNAC/(IX) group (Figure 1). In particular, the SNAC group (Figure 1) comprises several genes that regulate disease resistance pathways, as inferred from the increased resistance to pathogens upon overexpression under the control of a constitutive promoter. Data indicate that NAC TFs also have an important role in the regulation of plant defense responses to different pathogens in addition to wounding and insect feeding (Figure 5). The application of exogenous phytohormones, such as JA, SA, and ET, has also been shown to induce *NAC* genes in several species (Tran et al., 2004; He et al., 2005; Hu et al., 2006; Sindhu et al., 2008; Lu et al., 2007; Nakashima et al., 2007; Yokotani et al., 2009; Zheng et al., 2009; Xia et al., 2010a,b; Yoshii et al., 2010; Nuruzzaman et al., 2012b). Hence, NAC TFs can possibly modulate the phytohormonal regulation of the biotic stress cellular network for convergent and divergent adaptive pathways.

### NAC TFs in ROS and senescence signaling pathways

Reactive oxygen species (ROS) is an active molecule in most biotic plant stress. Such ROS as H_2_O_2_ act as important signal transduction molecules, mediating the acquisition of tolerance to various stresses (Bhattacharjee, 2005; Davletova et al., 2005). In rice, *OsNAC6* gene is involved in both response and tolerance to biotic stress (Nakashima et al., 2007). In Arabidopsis, ATAF subfamily (*ATAF1*, *ATAF2*, and *RD26*) is also involved in biotic stress. The expression of *RD26* is induced by JA and H_2_O_2_, and pathogen infections (Fujita et al., 2004; Zimmermann et al., 2004). Large-scale transcriptiome analysis with both types of mutants revealed that *RD26*-regulated genes are involved in the detoxification of ROS, defense, and senescence (Fujita et al., 2004; Balazadeh et al., 2011). The role of stress-responsive NAC proteins in senescence is poorly understood. Recently, the *NTL4*, (Lee et al., 2012), *MtNAC969* (de Zélicourt et al., 2012), *Os07g37920*, wheat *GPC* (Distelfeld et al., 2012) genes were found to be induced senescence in different plants. Leaf senescence is a unique developmental process that is characterized by massive programmed cell death and nutrient recycling. Leaf senescence is induced by pathogen infection (Dhindsa et al., 1981; Buchanan-Wollaston et al., 2003; Gepstein et al., 2003). *AtNAP* gene, which belongs to the closest NAC subfamily of the ATAF subfamily, has been shown to be involved in senescence (Guo and Gan, 2006). In addition all ATAF subfamily *NAC* genes, including *ATAF1*, *ATAF2*, and *RD26*, are upregulated during senescence in Arabidopsis leaves (Guo et al., 2004). These findings suggest that *RD26* may function at the node of convergence between the pathogen defense and senescence signaling pathways. Taken together, these results support the notion that ROS and senescence may be closely related to NAC-mediated stress responses.

## NAC function in abiotic stress

**Figure 6:**
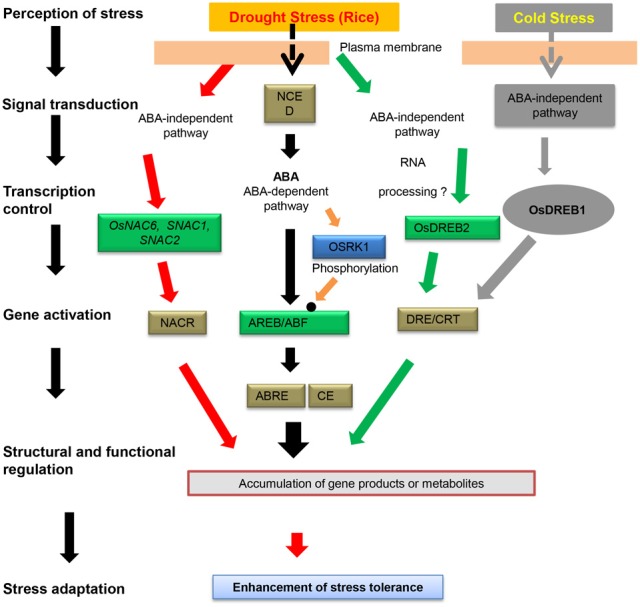
**Transcriptional regulatory networks of the *cis*-elements and NAC transcription factors involved in abiotic stress-induced gene expression in rice.** The *cis*-elements involved in stress-responsive transcription are shown in white boxes. TFs controlling stress-inducible gene expression are shown in green boxes. Protein kinases involved in the phosphorylation of TFs are shown in blue boxes. The small solid black circle indicates TF modification, i.e., through phosphorylation, in response to stress signals.

The NAC TFs function as important components in complex signaling progresses during plant stress responses. Considering the relatively large number of NAC TFs from different plants and their unknown and diverse roles under complex environmental stimuli, it remains a considerable challenge to uncover their roles in abiotic stress. Until recently, the possible involvement of TF NAC proteins in abiotic stress responses was deduced indirectly from transcription profiling; recent functional analyses, however, have provided some direct evidence. The recent data presented here mainly summarize the function of most NAC TFs in regulating the transcriptional reprogramming associated with plant abiotic responses (Figures 5, 6; Table 2). The tight regulation and fine-tuning of *NAC* genes during plant stress responses contribute to the establishment of complex signaling webs, and the important roles of *NAC* genes in plant abiotic stress responses make them potential candidates for imparting stress tolerance.

**Table 2:** **Function of NAC transcription factors in abiotic stresses**.

**Genes**	**Functions**	**Method**	**Species**	**References**
*ANAC019/AT1G52890*	Drought, high salinity, ABA signaling	Overexpression	*A. thaliana*	Tran et al., 2004
*ANAC055/AT3G15500*	Drought, high salinity, ABA signaling	Overexpression	*A. thaliana*	Tran et al., 2004
*ANAC072/AT4G27410*	Drought, high salinity, ABA signaling	Overexpression	*A. thaliana*	Tran et al., 2004
*RD26, RD20, Glyoxalase, Glutathione, transferase, Aldo/keto reductase, senesence associated gene13, cinnamil-alcohol dehydrogenase*	Drought, salt and ABA response	Overexpression	*A. thaliana*	Fujita et al., 2004
*ANAC019*, *COR47, RD29b, FER1, ERD11*	Cold, ABA signaling	Overexpression	*A. thaliana*	Jensen et al., 2010
*anac092-1, ANAC083, ANAC041, ANAC054, ANAC084*	Positive regulator of seed germination under salinity	Mutant	*A. thaliana*	Balazadeh et al., 2010
*ntl8-1*	Positive regulator of seed germination under salinity	Mutant	*A. thaliana*	Kim et al., 2008
*ATAF1*, *COR47*, *ERD10*, *KIN1*, *RD22*, *RD29A*	Positive regulator of drought tolerance	*knockouts (ataf1-1/2)*	*A. thaliana*	Lu et al., 2007
*ATAF1*, *ADH1*, *RD29A*, *COR47*	Positive regulator of drought tolerance	Overexpression	*A. thaliana*	Wu et al., 2009
*ONAC063*	Higher seed germination under high salinity and osmotic stress	Overexpression	*A. thaliana*	Yokotani et al., 2009
*AhNAC2*, *RD29A*, *RD29B*, *RAB18*, *ERD1*, *AtMYB2*, *AtMYC2*, *COR47*, *COR15a*, *KIN1*, *AREB1*, *CBF1*	Drought and salt tolerance	Overexpression	*A. thaliana*	Liu et al., 2011
*GmNAC20*, *DREB1A/CBF3*, *KIN2/cor6.6*, *Cor15A*, *RD29A/cor78*, *ARF19*, *LBD12*, *AIR1*	Salt and freezing tolerance	Overexpression	*G. max*, *A. thaliana*	Hao et al., 2011
*NTL8*	Salt tolerance, GA, and ABA pathway	Gene expression	*A. thaliana*	Kim et al., 2008
*ANAC019, ANAC055*	Defense disease, JA pathway	Overexpression	*A. thaliana*	Bu et al., 2008
*XND1*	Programmed cell death	Overexpression	*A. thaliana*	Zhao et al., 2008
*LOV1*	Cold response, photoperiod pathway	Overexpression	*A. thaliana*	Yoo et al., 2007
*NAC1*	Auxin, root development	Overexpression	*A. thaliana*	Guo et al., 2005
*ZmSNAC1*	Low temperature, high-salinity, drought stress, and abscisic acid (ABA)	Transgenic	*Z. mays*	Lu et al., 2012
*NTL6*, *SnRK2.8*	Drought-stress response	Overexpression/ RNAi	*A. thaliana*	Kim et al., 2012
*ANAC019*, *ANAC055* and *ANAC072*, *ICS1* and *BSMT1*	Inhibits salicylic acid accumulation	Transgenic	*A. thaliana*	Zheng et al., 2012
*TaNAC2*	Drought, salt, and freezing stresses	Overexpression	*A. thaliana*	Mao et al., 2012
*ANAC2/AT3G15510*	Salt and ABA stress tolerance	Overexpression	*A. thaliana*	He et al., 2005
*SNAC1/Os03g60080*	Stomata close, higher seed setting	Overexpression	*O. sativa*	Hu et al., 2006
*SNAC2/OsNAC6/Os01g66120*	Salt, drought, disease resistance drought, salinity, cold, wounding, and abscisic acid (ABA) treatment	Overexpression	*O. sativa*	Sindhu et al., 2008
*OsNAC5/ Os11g08210*	ABA, salt, cold tolerance, grain filling	Overexpression	*O. sativa*	Sperotto et al., 2009
*ONAC04/Os11g033005*	Drought, salt, cold tolerance	Overexpression	*O. sativa*	Zheng et al., 2009
*OsNAC10/Os11g03300*	Root, panicle, drought, salt, ABA	Overexpression	*O. sativa*	Jeong et al., 2010
*Ostil1*	Shoot branching	Overexpression	*O. sativa*	Mao et al., 2007
*RIM1*/*Os03g02800*	JA pathway signaling	Mutant	*O. sativa*	Yoshii et al., 2010
*Os07g04560, Os10g38834*	Root, severe drought	Microarray	*O. sativa*	Nuruzzaman et al., 2012b
*Os01g28050, Os01g29840*	Leaf, severe drought	Microarray	*O. sativa*	Nuruzzaman et al., 2012b
*Os03g12120*, *Os03g59730*, *Os06g15690*, *Os08g06140*, *Os08g33670*	Panicle, severe drought	Microarray	*O. sativa*	Nuruzzaman et al., 2012b
*Os12g41680*, *Os07g48550*, *Os11g03300*, *Os12g03040*, *Os01g66120*, *Os05g34830*,	Cold, drought, submergence, laidown-submergnece	Microarray	*O. sativa*	Nuruzzaman et al., 2010
*Os02g34970*, *Os07g48450*, *Os01g01430*, Os01g48460	Drought, submergence, laidown-submergnece	Microarray	*O. sativa*	Nuruzzaman et al., 2010
*OsOAT*, *SNAC2*	Drought and oxidative stress tolerance	Overexpression	*O. sativa*	You et al., 2013
*SNAC1*, *OsSRO1c*	Oxidative stress tolerance	Overexpression	*O. sativa*	You et al., 2013
*TaNAC69*	PEG-induced dehydration	Overexpression	*T*. *aestivum*	Xue et al., 2011
*GmNAC11*, *DREB1A*, *ERD11*, *Cor15A*, *ERF5*, *RAB18, KAT2*	Salt tolerance in soybean transgenic hairy roots	Overexpression	*G. max*	Hao et al., 2011
*GmNAC* glycoside hydrolases, defensins and glyoxalase I family proteins	Drought stress	Soybean array GeneChip	*G. max*	Le et al., 2011
*GmNAC085*	Dehydration stress	Soybean Affymetrix array	*G. max*	Le et al., 2011
*TaNAC2a*	Drought tolerance	Overexpression	*N. tabacum*	Tang et al., 2012
*DgNAC1*	ABA, NaCl, drought and cold	Overexpression	*N. tabacum*	Liu et al., 2011
*CarNAC3*	Seed germination, drought, ethephon, ABA, IAA signaling	Transcriptome	*C. arietinum*	Peng et al., 2009
*miR319, AsNAC60*	Drought and salinity stress		*Agrostis stolonifera*	Zhou et al., 2013
*EcNAC1*	Water-deficit and salt stress	Overexpression	*N. tabacum*	Ramegowda et al., 2012
*AhNAC2*	Salt	Overexpression	Arachis	Liu et al., 2011
*RhNAC2* or *RhEXPA4*	Dehydration tolerance	Transgenic	*R. hybrida*	Dai et al., 2012
*ClNAC*	Hormonal treatments including salt, drought, cold, heat, abscisic acid and salicylic acid treatments	Reverse transcriptase polymerase chain reaction	*C. lavandulifolium*	Huang et al., 2012
*CsNAM*	Drought, osmoticum, salt, heat and hydrogen peroxide		*Camellia sinensis*	Paul et al., 2012
*Os04g0477300*	Boron-toxicity tolerance	RNA interference	*O. sativa*	Ochiai et al., 2011
*SiNAC*	Dehydration, salinity, ethephon, and methyl jasmonate.	Transcription	*S. italica*	Puranik et al., 2011
*ANAC102*	Waterlogging	Overexpression	*A. thaliana*	Christianson et al., 2009
*HSImyb* and *HSINAC*	Gibberellin response	Transcript	*H. vulgare*	Robertson, 2004
*ANAC042* it is also in biotic	Heat stress	Overexpression	*A. thaliana*	Shahnejat-Bushehri et al., 2012
*TaNAC2a*, *TaNAC4a*, *TaNAC6*, *TaNAC7*, *TaNAC13* and *TaNTL5*	Dehydration, salinity and low temperature	Transgenic	*T. aestivum*	Tang et al., 2012
*TaNAC4*	Environmental stimuli, including high salinity, wounding, and low-temperature also induced	Transcription	*T. aestivum*	Xia et al., 2010a
*ONAC063*	High-temperature and high-salinity	Transactivation	*O. sativa*	Yokotani et al., 2009

### Drought, salinity, cold, and osmotic stress

Abiotic stress triggers a wide range of plant responses, from the alteration of gene expression and cellular metabolism to changes in plant growth, development, and crop yield. Thus, understanding the complex mechanism of drought and salinity tolerance is important for agriculture production. Interestingly, many *NAC* genes have been shown to be involved in plant responses to drought and salinity stress. In transgenic rice, the *Os01g66120/OsNAC2/6* and *Os11g03300/OsNAC10* genes were found to enhance drought and salt tolerance (Figure 5; Nakashima et al., 2009; Jeong et al., 2010), and *Os03g60080/SNAC1* increased grain yield (21–34%) under drought stress (Hu et al., 2006). Udupa et al. (1999) reported that comparative gene expression profiling is an efficient way to identify the pathways and genes regulating a stress response under different stress conditions. The Arabidopsis *NAC* gene *ANAC092* demonstrates an intricate overlap of *ANAC092*-mediated gene regulatory networks during salt-promoted senescence and seed maturation (Balazadeh et al., 2010). Lan et al. (2005) found that a large portion of the genes regulated by dehydration are also up-regulated by fertilization; indeed, pollen is a major site of variations in the expression levels for many genes (Czechowski et al., 2005). Related conclusions have been drawn from analyses based on promoter-GUS fusions of cold-inducible *Os01g66120/SNAC2/6*, *Os11g03300/OsNAC10*, *RD29A*, *COR15A*, *KIN1*, and *COR6.6* in rice and Arabidopsis, genes that are regulated during plant development (root, leaf, and pollen) under both stress (drought and cold) and non-stress conditions (Sindhu et al., 2008; Jeong et al., 2010). You et al. (2013) reported that *OsOAT* is a direct target of the stress-responsive NAC transcription factor SNAC2, and *OsOAT* overexpression in rice resulted in significantly enhanced drought and osmotic stress tolerance. Plants overexpressing *GmNAC085* show enhanced drought tolerance (Le et al., 2011), whereas the overexpression of *GmNAC11* led to increased sensitivity to salt and mannitol stresses (Hao et al., 2011). Microarray profiling of the roots and leaves of drought-treated rice revealed the induction of 17 *NAC* genes by severe or mild drought treatment (Nuruzzaman et al., 2012b). *SiNAC* is also simultaneously induced by dehydration, salinity, ethephon, and methyl jasmonate treatments (Puranik et al., 2011). Similarly, the expression of *DgNAC1, TaNAC2a* and *EcNAC1* were strongly induced by NaCl and drought stresses in transgenic tobacco plants (Liu et al., 2011; Ramegowda et al., 2012; Tang et al., 2012). Several genes, such as *ZmSNAC1* (Lu et al., 2012), *TaNAC69* (Xue et al., 2011), *CarNAC3* (Peng et al., 2009), *miR319, AsNAC60* (Zhou et al., 2013), *AhNAC2* (Liu et al., 2011), *RhNAC2* or *RhEXPA4* (Dai et al., 2012), *ClNAC* (Huang et al., 2012), *CsNAM* (Paul et al., 2012), *SiNAC* (Puranik et al., 2011), *HSImyb* and *HSINAC* (Robertson, 2004), and *TaNAC2a, TaNAC4a, TaNAC6*, and *TaNAC4* (Tang et al., 2012; Xia et al., 2010a), were increased by drought and NaCl (Figure 5; Table 2).

### Phytohormone signaling pathway

The expression of members of the *OsNAC* gene family under hormone treatment requires extensive cross-talk between the response pathways, and it is likely that substantial physiological connections exist between NAC protein production and phytohormone treatment. Phytohormones are involved in influencing signaling responses by acting in conjunction with or in opposition to each other to maintain cellular homeostasis (Fujita et al., 2006; Miller et al., 2008). The NAC TFs form a complex but interesting group of important arbitrators of this process (Figure 5). *ANAC019* and *ANAC055* are involved in both ABA- and JA-mediated regulation (Greve et al., 2003; Bu et al., 2008, 2009; Jensen et al., 2010). The ATAF subfamily TFs are another group of NAC proteins that act at the convergence point of biotic and abiotic stress signaling (Delessert et al., 2005; He et al., 2005; Jensen et al., 2007). Because *ATAF1* alleles expedite drought perception at the cost of optimal basal defense, ATAF1 acts as a negative regulator of ABA signaling but induces JA/ET-associated defense signaling marker genes (Jensen et al., 2008). Conversely, *ATAF2* expression was induced by dehydration, JA, and SA (Figure 5; Delessert et al., 2005). We have proposed the participation of *SiNAC* in the ABA-independent pathway of abiotic stress and in regulating biotic stress via an antagonistic JA and SA pathway (Puranik et al., 2011). A number of *NAC* genes (e.g., *AtNAC2*) in plants are affected by auxin, ethylene (Xie et al., 2000; He et al., 2005), and ABA (e.g., *OsNAC5*; Sperotto et al., 2009). In Arabidopsis, NAC TF *NTL8* regulates GA3-mediated salt signaling in seed germination (Kim et al., 2008). ABA plays a major role in mediating the adaptation of a plant to stress, and this hormone can stimulate root growth in plants that need to increase their ability to extract water from the soil. *OsNAC5*/*ONAC009*/*ONAC071* and *OsNAC6* are homologs that are induced by abiotic stress, such as drought and high salinity, and ABA (Takasaki et al., 2010). *AtNAC1* and *AtNAC2* are induced by auxin and ABA, respectively, and *AtNAC1* mediates auxin signaling to promote lateral root development in Arabidopsis (Xie et al., 2000; He et al., 2005). ABA signaling induces the expression of genes encoding proteins that protect the cells in vegetative tissues from damage when they become dehydrated. These well-known ABA responses are less sensitive to ABA in NPX1-overexpressing plants (Kim et al., 2009). The expression of the *RD26* gene is induced by drought and also ABA and high salinity (Fujita et al., 2004). NAC TFs regulate many target genes by binding to the CATGTG motif in the promoter region of the target gene to activate transcription in the response to drought stress (Nakashima et al., 2007), a transcriptional regulatory system that is known as a regulon. ABA is produced under conditions of drought stress and plays a crucial role in drought tolerance in plants (Figure 6; Shinozaki et al., 2003). In addition to NAC and other regulons, *OsDREB2* responds to dehydration in rice (Dubouzet et al., 2003); the dehydration-responsive element binding protein 1 (DREB1)/C-repeat-binding factor (CBF) and DREB2 regulons function in ABA-independent gene expression, whereas the ABA-responsive element (ABRE)-binding protein (AREB)/ABRE-binding factor (ABF) regulon functions in ABA-dependent gene expression. ABA-activated OSRK1 protein kinases phosphorylate and activate AREB/ABF-type proteins in rice (Figure 6; Chae et al., 2007). Both ABA-independent and ABA-dependent signal transduction pathways convert the initial stress signal into cellular responses (Figures 5, 6). The TF family members involved in both ABA-independent (AP2/ERF, bHLH, and NAC) and ABA-dependent (MYB, bZIP, and MYC) pathways are up-regulated in rice; the TFs belonging to this family interact with specific *cis*-elements and/or proteins, and their overexpression confers stress tolerance in heterologous systems (Fujita et al., 2004; Tran et al., 2004; Hu et al., 2006). The expression of *OsNAC6* is induced by ABA and abiotic stresses, including cold, drought, and high salinity (Nakashima et al., 2007). Together, these data provide evidence that different *NAC* genes play differential roles in the specific responses to different phytohormone treatments. Thus, gene expression profiles under both biotic and abiotic stresses to determine the vital role of *NAC* genes in plant growth and stress responses and the identification of target genes for TFs involved in stress responses are important.

### Temperature stress

In agriculture, high or low temperature acts as a major negative factor limiting crop production. Indeed, tremendous work has been performed in the past two decades to reveal the complex molecular mechanism in the plant responses to extreme temperature, and there is increasing evidence that NAC proteins are involved in responses to both heat and cold stresses. For example, an NAC TF gene (*ONAC063*) in rice roots responds to a combination of high-temperature stress (Yokotani et al., 2009). Another example is that transgenic Arabidopsis plants overexpressing *ANAC042* show increased tolerance to heat stress when compared to the wild-type plants (Shahnejat-Bushehri et al., 2012). Moreover, the overexpression of *ZmSNAC1* enhanced the tolerance to drought and low-temperature stress compared to the control (Lu et al., 2012). The expression of *OsNAC10*, *SNAC2/OsNAC6*, *TaNAC4,NTL6, TaNAC2a, TaNAC4a, TaNAC6,TaNAC7,TaNAC13*, and *TaNTL5* is induced by low temperature in plants (Jeong et al., 2010; Xia et al., 2010a; Tang et al., 2012), and a gene expressing a CsNAM-like protein is induced by heat in tea plants (Paul et al., 2012). Northern blot and SNAC2 promoter activity analyses suggest that the *SNAC2* gene is induced by low temperature. Additionally, a microarray analysis of rice *NAC* genes has revealed that 8 of the 14 analyzed *OsNAC* genes are regulated by severe or mild drought stress (Nuruzzaman et al., 2012b), showing distinct expression patterns upon high-temperature treatment. Yoo et al. (2007) reported that the phenotype resulting from the overexpression of an NAC-domain protein gene (*At2g02450*) is related to the control of flowering time and cold responses. The importance of NAC proteins in plant development, transcription regulation, and regulatory pathways involving protein–protein interactions is being increasingly recognized. Taken together, NAC proteins function in plants adaptions to temperature variations through the transcriptional reprogramming of downstream stress-related genes.

### Nutrient-use efficiency

Various nutrient elements are required for the normal growth and development of plants. Boron (B) is an essential micronutrient for higher plants, but excessive amounts of B inhibit growth (B toxicity). As the optimal range of B concentration in tissues is narrow (Blamey et al., 1997), B toxicity occurs in many plants at levels only slightly above that required for normal growth (Mengel and Kirkby, 2001). The *Os04g0477300* gene encodes an NAC-like TF, and the function of the transcript is abolished in B toxicity-tolerant cultivars. Transgenic plants in which the expression of *Os04g0477300* is abolished by RNA interference acquire a tolerance to B toxicity (Ochiai et al., 2011). In a transcriptome analysis using Arabidopsis plants under B toxicity, nine genes encoding multidrug and toxic compound extrusion transporters, a zinc-finger family TF, a heat-shock protein-like protein, an NAC-like TF, and unknown proteins were induced (Kasajima and Fujiwara, 2007), though the functions of these proteins are not yet known. A sufficient supply of inorganic phosphate (Pi) is vital to plants, and the low bioavailability of Pi in soils is often a limitation to growth and development. Consequently, plants have evolved a range of regulatory mechanisms to adapt to phosphorus-starvation to optimize the uptake and assimilation of Pi. Recently, significant progress has been achieved in elucidating these mechanisms, revealing that the coordinated expression of a large number of genes is important for many of these adaptations. These studies provide a valuable basis for the identification of new regulatory genes and promoter elements to further the understanding of Pi-dependent gene regulation. With a focus on the Arabidopsis transcriptome, Nilsson et al. (2010) reported common findings that indicate new groups of putative regulators, including the NAC, MYB, and WRKY families. With a number of new discoveries of regulatory elements, a complex regulatory network is emerging. They evaluate the contribution of the regulatory elements to P-responses and present a model comprising the factors directly or indirectly involved in transcriptional regulation. Thus, *NAC* genes appear to respond to several aspects of nutrient excess and deficiency-induced stresses, implicating their diverse functions in these signaling pathways.

## One NAC for multiple processes

Numerous studies have demonstrated that a single TF may function in several seemingly disparate signaling pathways, as can be deduced from their induced expression profiles by various stress factors. *OsNAC6* was induced by JA, a plant hormone that activates defense responses against herbivores and pathogens (Figures 5, 6; Ohnishi et al., 2005). Studies on an *NAC* gene (*Os04g0477300*) showed that it functions in at least three different processes, including pathogen defense, senescence, and responses to phosphate and boron deficiency (Uauy et al., 2006; Waters et al., 2009; Nilsson et al., 2010; Ochiai et al., 2011). A number of *NAC* genes (e.g., *AtNAC2*) in plants are affected by auxin, ethylene (Xie et al., 2000; He et al., 2005), and ABA (e.g., *OsNAC5*; Sperotto et al., 2009). *Os05g34830* (SNAC group, Figure 1) was specifically induced in the roots of a tolerant line under severe and mild drought conditions and was activated by ABA treatment (Nuruzzaman et al., 2012b). *OsNAC5*/*ONAC009*/*ONAC071* and *OsNAC6* are homologs that are induced by pathogen infection and such abiotic stresses as drought and high salinity and ABA (Takasaki et al., 2010). *AtNAC1* and *AtNAC2* are induced by auxin and ABA, respectively, and *AtNAC1* mediates auxin signaling to promote lateral root development in Arabidopsis (Xie et al., 2000; He et al., 2005). The *TaNAC4* gene functions as a transcriptional activator involved in wheat responses to abiotic and biotic stresses (Xia et al., 2010a). *SiNAC* transcripts mostly accumulate in young spikes and were strongly induced by dehydration, salinity, ethephon, and methyl jasmonate (Distelfeld et al., 2012). These data demonstrate that a single *NAC* gene can function as regulator of several different processes and may also mediate the cross-talk between different signaling pathways.

## Conclusion

The responses to the environment are specialized through the diversification of the structure of stress-response regulators, which are involved in stress-response pathways via binding motifs (CATGTG) in their target genes. Thus, the components and regulatory structure of specific pathways must be delimited for an understanding of the evolutionary genetics of environmental stress responses. This review summarizes the current knowledge of the genes and NAC TFs that comprise a portion of this network. Interestingly, all of the SNAC sequences known to play a role in disease resistance responses are in one group of the NAC family. Much progress in NAC TF functional research has been attained over the past decade. However, most of these advances are related to the involvement of biotic stress. The identification of NAC functions in biotic and abiotic stresses will remain a substantial challenge in the coming years. To achieve a better understanding of their role during both types of stress, it is very important to identify the interacting partner of NAC proteins that cooperates in regulating the transcription of downstream target genes under a specific condition. It is also crucial to identify the key components of the signal transduction pathways with which these factors physically interact. Applying data obtained from microarrays could help to directly determine the specific NAC DNA-binding sites on a global scale under conditions of biotic and abiotic stress. Accordingly, we may then appreciate the complex mechanisms of signaling and transcriptional reprogramming controlled by NAC proteins and the plant processes in which they participate. Certainly, further molecular studies of NAC NFs under different stresses will clarify the fine-tuning mechanisms that are controlled by NAC proteins in plants, with economical benefits to agricultural production.

### Conflict of interest statement

The authors declare that the research was conducted in the absence of any commercial or financial relationships that could be construed as a potential conflict of interest.

## References

[B1] Aida M. Ishida T. Fukaki H. Fujisawa H. Tasaka M. (1997). Genes involved in organ separation in *Arabidopsis*: an analysis of the cup-shaped cotyledon mutant. Plant Cell 9, 841–857 10.1105/tpc.9.6.841 9212461 PMC156962

[B2] Balazadeh S. Kwasniewski M. Caldana C. Mehrnia M. Zanor M. I. Xue G. P. (2011). ORS1, an H2O2-responsive NAC transcription factor, controls senescence in *Arabidopsis thaliana*. Mol. Plant 4, 346–360 10.1093/mp/ssq080 21303842 PMC3063519

[B3] Balazadeh S. Siddiqui H. Allu A. D. Matallana-Ramirez L. P. Caldana C. Mehrnia M. (2010). A gene regulatory network controlled by the NAC transcription factor ANAC092/AtNAC2/ORE1 during salt-promoted senescence. Plant J. 62, 250–264 10.1111/j.1365-313X.2010.04151.x 20113437

[B4] Bhattacharjee S. (2005). Reactive oxygen species and oxidative burst: roles in stress, senescence and signal transduction in plants. Curr. Sci. 89, 1113–1121

[B5] Biruma M. Martin T. Fridborg I. Okori P. Dixelius C. (2012). Two loci in sorghum with NB-LRR encoding genes confer resistance to *Colletotrichum sublineolum*. Theor. Appl. Genet. 124, 1005–1015 10.1007/s00122-011-1764-8 22143275

[B6] Blamey F. P. C. Asher C. J. Edwards D. G. (1997). Boron deficiency in sunflower, in Boron in Soils and Plants, eds Bell R. W. Rerkasem B. (Dordrecht: Kluwer), 145–149

[B7] Breeze E. Harrison E. McHattie S. Hughes L. Hickman R. Hill C. (2011). High-resolution temporal profiling of transcripts during *Arabidopsis* leaf senescence reveals a distinct chronology of processes and regulation. Plant Cell 23, 873–894 10.1105/tpc.111.083345 21447789 PMC3082270

[B8] Bu Q. Jian H. Li C. B. Zhai Q. Zhang J. Wu X. (2008). Role of the *Arabidopsis thaliana* NAC transcription factors ANAC019 and ANAC055 in regulating jasmonic acid signaled defense responses. Cell Res. 18, 756–767 10.1038/cr.2008.53 18427573

[B9] Bu Q. Li H. Zhao Q. Jiang H. Zhai Q. Zhang J. (2009). The Arabidopsis RING finger E3 ligase RHA2a is a novel positive regulator of abscisic acid signaling during seed germination and early seedling development. Plant Physiol. 150, 463–481 10.1104/pp.109.135269 19286935 PMC2675735

[B10] Buchanan-Wollaston V. Earl S. Harrison E. Mathas E. Navabpour S. Page T. (2003). The molecular analysis of leaf senescence–a genomics approach. Plant Biotechnol. J. 1, 3–22 10.1046/j.1467-7652.2003.00004.x 17147676

[B11] Chae M. J. Lee J. S. Nam M. H. Cho K. Hong J. Y. Yi S. A. (2007). A rice dehydration-inducible SNF1 related protein kinase 2 phosphorylates an abscisic acid responsive element binding factor and associates with ABA signaling. Plant Mol. Biol. 63, 151–169 10.1007/s11103-006-9079-x 16977424

[B12] Chakravarthy S. Vela0squez A. C. Ekengren S. K. Collmer A. Martin G. B. (2010). Identification of *Nicotiana benthamiana* genes involved in pathogen-associated molecular pattern-triggered immunity. Mol. Plant Microbe Interact. 23, 715–726 10.1094/MPMI-23-6-0715 20459311

[B13] Chen Q. Wang Q. Xiong L. Lou Z. (2011). A structural view of the conserved domain of rice stress-responsive NAC1. Protein Cell 2, 55–63 10.1007/s13238-011-1010-9 21337010 PMC4875291

[B14] Chern M. S. Fitzgerald H. A. Canlas P. E. Navarre D. A. Ronald P. C. (2005). Over-expression of rice NPR1 homologue leads to constitutive activation of defense response and hypersensitivity to light. Mol. Plant Microbe Interact. 18, 511–520 10.1094/MPMI-18-0511 15986920

[B15] Christianson J. A. Wilson I. W. Llewellyn D. J. Dennis E. S. (2009). The lowoxygen induced NAC domain transcription factor ANAC102 affects viability of *Arabidopsis thaliana* seeds following low-oxygen treatment. Plant Physiol. 149, 1724–1738 10.1104/pp.108.131912 19176720 PMC2663757

[B16] Collinge M. Boller T. (2001). Differential induction of two potato genes, *Stprx2* and *StNAC*, in response to infection by *Phytophthora infestans* and to wounding. Plant Mol. Biol. 46, 521–529 10.1023/A:1010639225091 11516145

[B17] Czechowski T. Stitt M. Altmann T. Udvardi M. K. Scheible W. R. (2005). Genome-wide identification and testing of superior reference genes for transcript normalization in Arabidopsis. Plant Physiol. 139, 5–17 10.1104/pp.105.063743 16166256 PMC1203353

[B18] Dai F. Zhang C. Jiang X. Kang M. Yin X. Lü P. (2012). *RhNAC2* and *RhEXPA4* are involved in the regulation of dehydration tolerance during the expansion of rose petals. Plant Physiol. 160, 2064–2082 10.1104/pp.112.207720 23093360 PMC3510132

[B19] Davletova S. Rizhsky L. Liang H. J. Zhong S. Q. Oliver D. J. Coutu J. (2005). Cytosolic ascorbate peroxidase 1 is a central component of the reactive oxygen gene network of *Arabidopsis*. Plant Cell 17, 268–281 10.1105/tpc.104.026971 15608336 PMC544504

[B20] de Zélicourt A. Diet A. Marion J. Laffont C. Ariel F. Moison M. (2012). Dual involvement of a *Medicago truncatula* NAC transcription factor in root abiotic stress response and symbiotic nodule senescence. Plant J. 70, 220–230 10.1111/j.1365-313X.2011.04859.x 22098255

[B21] Delessert C. Kazan K. Wilson I. W. Van Der Straeten D. Manners J. Dennis E. S. (2005). The transcriptionfactor ATAF2 represses the expression of pathogenesis-related genes in Arabidopsis. Plant J. 43, 745–757 10.1111/j.1365-313X.2005.02488.x 16115070

[B22] Dhindsa R. S. Plumb-Dhindsa P. Thorpe T. A. (1981). Leaf senescence: correlated with increased levels of membrane permeability and lipid peroxidation and decreased levels of superoxide dismutase and catalase. J. Exp. Bot. 32, 93–101 10.1093/jxb/32.1.93

[B23] Ding X. S. Schneider W. L. Chaluvadi S. R. Mian M. A. Nelson R. S. (2006). Characterization of a Brome mosaic virus strain and its use as a vector for gene silencing in monocotyledonous hosts. Mol. Plant Microbe Interact. 19, 1229–1239 10.1094/MPMI-19-1229 17073305

[B24] Distelfeld A. Pearce S. P. Avni R. Scherer B. Uauy C. Piston F. (2012). Divergent functions of orthologous NAC transcription factors in wheat and rice. Plant Mol. Biol. 78, 515–524 10.1007/s11103-012-9881-6 22278768 PMC4773031

[B25] Dubouzet J. G. Sakuma Y. Ito Y. Kasuga M. Dubouzet E. G. Miura S. (2003). *OsDREB* genes in rice, *Oryza sativa* L, encode transcription activators that function in drought, high salt and cold-responsive gene expression. Plant J. 33, 751–763 10.1046/j.1365-313X.2003.01661.x 12609047

[B26] Duval M. Hsieh T. F. Kim S. Y. Thomas T. L. (2002). Molecular characterization of AtNAM: a member of the Arabidopsis NAC domain super family. Plant Mol. Biol. 50, 237–248 10.1023/A:1016028530943 12175016

[B27] Ernst H. A. Olsen A. N. Larsen S. Lo-Leggio L. (2004). Structure of the conserved domain of ANAC, a member of the NAC family of transcription factors, EMBO Rep. 5, 297–303 15083810 10.1038/sj.embor.7400093 PMC1299004

[B28] Eulgem T. Somssich I. E. (2007). Networks of WRKY transcription factors in defense signaling. Curr. Opin. Plant Biol. 10, 366–371 10.1016/j.pbi.2007.04.020 17644023

[B29a] Faria J. A. Reis P. A. Reis M. T. Rosado G. L. Pinheiro G. L. Mendes G. C. (2011). The NAC domain-containing protein, GmNAC6, is a downstream component of the ER stress- and osmotic stress-induced NRP-mediated cell-death signaling pathway. BMC Plant Biol. 11:129 10.1186/1471-2229-11-129 21943253 PMC3193034

[B29] Fujita M. Fujita Y. Maruyama K. Seki M. Hiratsu K. Ohme-Takagi M. (2004). A dehydration-induced NAC protein, RD26, is involved in a novel ABA-dependent stress-signaling pathway. Plant J. 39, 863–876 10.1111/j.1365-313X.2004.02171.x 15341629

[B30] Fujita M. Fujita Y. Noutoshi Y. Takahashi F. Narusaka Y. Yamaguchi-Shinozaki K. (2006). Crosstalk between abiotic and biotic stress responses: a current view from the points of convergence in the stress signaling networks. Curr. Opin. Plant Biol. 9, 436–442 10.1016/j.pbi.2006.05.014 16759898

[B31] Gepstein S. Sabehi G. Carp M. J. Hajouj T. Nesher M. F. Yariv I. (2003). Large-scale identification of leaf senescence-associated genes. Plant J. 36, 629–642 10.1046/j.1365-313X.2003.01908.x 14617064

[B32] Greve K. La Cour T. Jensen M. K. Poulsen F. M. Skriver K. (2003). Interactions between plant RING-H2 and plant specific NAC (NAM/ATAF1/2/CUC2) proteins: RING-H2 molecular specificity and cellular localization. Biochem. J. 371, 97–108 10.1042/BJ20021123 12646039 PMC1223272

[B33] Guo H. S. Xie Q. Fei J. F. Chua N. H. (2005). Micro RNA directs mRNA cleavage of the transcription factor NAC1 to down-regulate auxin signals for Arabidopsis lateral root development. Plant Cell 17, 1376–1386 10.1105/tpc.105.030841 15829603 PMC1091761

[B34] Guo Y. Gan S. (2006). AtNAP, a NAC family transcription factor, has an important role in leaf senescence. Plant J. 46, 601–612 10.1111/j.1365-313X.2006.02723.x 16640597

[B35] Guo Y. Cai Z. Gan S. (2004). Transcriptome of Arabidopsis leaf senescence. Plant Cell Environ. 27, 521–549 10.1111/j.1365-3040.2003.01158.x

[B36] Gutterson N. Reuber T. L. (2004). Regulation of disease resistance pathways by AP2/ERF transcription factors. Curr. Opin. Plant Biol. 7, 465–471 10.1016/j.pbi.2004.04.007 15231271

[B37] Hao Y. J. Wei W. Song Q. X. Chen H. W. Zhang Y. Q. Wang F. (2011). Soybean NAC transcription factors promote abiotic stress tolerance and lateral rootformation in transgenic plants. Plant J. 68, 302–313 10.1111/j.1365-313X.2011.04687.x 21707801

[B38] He X. J. Mu R. L. Cao W. H. Zhang Z. G. Zhang J. S. Chen S. Y. (2005). AtNAC2, a transcription factor downstream of ethylene and auxin signaling pathways, is involved in salt stress response and lateral root development. Plant J. 44, 903–916 10.1111/j.1365-313X.2005.02575.x 16359384

[B39] Hegedus D. Yu M. Baldwin D. Gruber M. Sharpe A. Parkin I. (2003). Molecular characterization of *Brassica napus* NAC domain transcriptional activators induced in response to biotic and abiotic stress. Plant Mol. Biol. 53, 383–397 10.1023/B:PLAN.0000006944.61384.11 14750526

[B40] Hein I. Barciszewska-Pacak M. Hrubikova K. Williamson S. Dinesen M. Soenderby I. E. (2005). Virus-induced gene silencing-based functional characterization of genes associated with powdery mildew resistance in barley. Plant Physiol. 138, 2155–2164 10.1104/pp.105.062810 16040663 PMC1183403

[B41] Hu H. Dai M. Yao J. Xiao B. Li X. Zhang Q. (2006). Overexpressing a NAM, ATAF, and CUC (NAC) transcription factor enhances drought resistance and salt tolerance in rice. Proc. Natl. Acad. Sci. U.S.A. 103, 12987–12992 10.1073/pnas.0604882103 16924117 PMC1559740

[B42] Hu H. You J. Fang Y. Zhu X. Qi Z. Xiong L. (2008). Characterization of transcription factor gene *SNAC2* conferring cold and salt tolerance in rice. Plant Mol. Biol. 67, 169–181 10.1007/s11103-008-9309-5 18273684

[B43] Hu R. Qi G. Kong Y. Kong D. Gao Q. Zhou G. (2010). Comprehensive analysis of NAC domain transcription factor gene family in *Populus trichocarpa*. BMC Plant Biol. 10:145 10.1186/1471-2229-10-145 20630103 PMC3017804

[B44] Huang H. Wang Y. Wang S. Wu X. Yang K. Niu Y. (2012). Transcriptome-wide survey and expression analysis of stress-responsive NAC genes in *Chrysanthemum lavandulifolium*. Plant Sci. 194, 18–27 10.1016/j.plantsci.2012.05.004 22794915

[B45] Huh S. U. Lee S. B. Kim H. H. Paek K. H. (2012). ATAF2, a NAC transcription factor, binds to the promoter and regulates *NIT2* gene expression involved in auxin biosynthesis. Mol. Cells 34, 305–313 10.1007/s10059-012-0122-2 22965747 PMC3887843

[B46] Jensen M. K. Hagedorn P. H. de Torres-Zabala M. Grant M. R. Rung J. H. Collinge D. B. (2008). Transcriptional regulationbyanNAC (NAMATAF1,2-CUC2) transcription factor attenuates ABA signaling for efficient basal defence towards *Blumeria graminis* f sp *hordei* in Arabidopsis. Plant J. 56, 867–880 10.1111/j.1365-313X.2008.03646.x 18694460

[B47] Jensen M. K. Kjaersgaard T. Nielsen M. M. Galberg P. Petersen K. O'Shea C. (2010). The *Arabidopsis thaliana* NAC transcription factor family: structure-function relationships and determinants of ANAC019 stress signaling. Biochem. J. 426, 183–196 10.1042/BJ20091234 19995345

[B48] Jensen M. K. Rung J. H. Gregersen P. L. Gjetting T. Fuglsang A. T. Hansen M. (2007). The HvNAC6 transcription factor: a positive regulator of penetration resistance in barley and Arabidopsis. Plant Mol. Biol. 65, 137–150 10.1007/s11103-007-9204-5 17619150

[B49] Jeong J. S. Kim Y. S. Baek K. H. Jung H. Ha S. H. Do Choi, Kim M. (2010). Root-specific expression of *OsNAC10* improves drought tolerance and grain yield in rice under field drought conditions. Plant Physiol. 153, 185–197 10.1104/pp.110.154773 20335401 PMC2862432

[B50] Jeong R. D. Chandra-Shekara A. C. Kachroo A. Klessig D. F. Kachroo P. (2008). HRT-mediated hypersensitive response and resistance to Turnip crinkle virus in Arabidopsis does not require the function of TIP, the presumed guardee protein. Mol. Plant Microbe Interact. 21, 1316–1324 10.1094/MPMI-21-10-1316 18785827

[B51] Kaneda T. Taga Y. Takai R. Iwano M. Matsui H. Takayama S. (2009). The transcription factor OsNAC4 is a key positive regulator of plant hypersensitive cell death. EMBO J. 28, 926–936 10.1038/emboj.2009.39 19229294 PMC2670867

[B52] Kasajima I. Fujiwara T. (2007). Identification of novel *Arabidopsis thaliana* genes which are induced by high levels of boron. Plant Biotechnol. 24, 355–360 10.5511/plantbiotechnology.24.355

[B53] Kim J. H. Woo H. R. Kim J. Lim P. O. Lee I. C. Choi S. H. (2009). Trifurcate feed-forward regulation of age-dependent cell death involving miR164 in Arabidopsis. Science 323, 1053–1057 10.1126/science.1166386 19229035

[B54] Kim M. J. Park M. J. Seo P. J. Song J. S. Kim H. J. Park C. M. (2012). Controlled nuclear import of the transcription factor NTL6 reveals a cytoplasmic role of SnRK2.8 in the drought-stress response. Biochem. J. 448, 353–363 10.1042/BJ20120244 22967043

[B55] Kim S. G. Kim S. Y. Park C. M. (2007a). A membrane-associated NAC transcription factor regulates salt-responsive flowering via FLOWERING LOCUS T in Arabidopsis. Planta 226, 647–654 17410378 10.1007/s00425-007-0513-3

[B56] Kim S. Y. Kim S. G. Kim Y. S. Seo P. J. Bae M. Yoon H. K. (2007b). Exploring membrane-associated NAC transcription factors in Arabidopsis: implications for membrane biology in genome regulation. Nucleic Acids Res. 35, 203–213 17158162 10.1093/nar/gkl1068 PMC1802569

[B57] Kim S. G. Lee A. K. Yoon H. K. Park C. M. (2008). A membrane-bound NAC transcription factor NTL8 regulates gibberellic acid-mediated salt signaling in Arabidopsis seed germination. Plant J. 55, 77–88 10.1111/j.1365-313X.2008.03493.x 18363782

[B58] Kim Y. S. Kim S. G. Park J. E. Park H. Y. Lim M. H. Chua N. H. (2006). A membrane-bound NAC transcription factor regulates cell division in Arabidopsis. Plant Cell 18, 3132–3144 10.1105/tpc.106.043018 17098812 PMC1693948

[B59] Ko J. H. Yang S. H. Park A. H. Lerouxel O. Han K. H. (2007). ANAC012, a member of the plant-specific NAC transcription factor family, negatively regulates xylary fiber development in *Arabidopsis thaliana*. Plant J. 50, 1035–1048 10.1111/j.1365-313X.2007.03109.x 17565617

[B60] Kranz H. D. Denekamp M. Greco R. Jin H. Leyva A. Meissner R. C. (1998). Towards functional characterisation of the members of the R2R3-MYB gene family from *Arabidopsis thaliana*. Plant J. 16, 263–276 10.1046/j.1365-313x.1998.00278.x 9839469

[B61] Lan L. Li M. Lai Y. Xu W. Kong Z. Ying K. (2005). Microarray analysis reveals similarities and variations in genetic programs controlling pollination/fertilization and stress responses in rice (*Oryza sativa* L.). Plant Mol. Biol. 59, 151–164 16217609 10.1007/s11103-005-3958-4

[B62] Le D. T. Nishiyama R. Watanabe Y. Mochida K. Yamaguchi-Shinozaki K. Shinozaki K. (2011). Genome-wide survey and expression analysis of the plant-specific NAC transcription factor family in soybean during development and dehydration stress. DNA Res. 18, 263–276 10.1093/dnares/dsr015 21685489 PMC3158466

[B63] Lee S. Seo P. J. Lee H. J. Park C. M. (2012). A NAC transcription factor NTL4 promotes reactive oxygen species production during drought-induced leaf senescence in Arabidopsis. Plant J. 70, 831–844 10.1111/j.1365-313X.2012.04932.x 22313226

[B64] Li W. Zhong S. Li G. Li Q. Mao B. Deng Y. (2011). Rice RING protein OsBBI1 with E3 ligase activity confers broad-spectrum resistance against *Magnaporthe oryzae* by modifying the cell wall defence. Cell Res. 21, 835–848 10.1038/cr.2011.4 21221134 PMC3203666

[B65] Lin R. Zhaom W. Mengm X. Wang M. Peng Y. (2007). Rice gene *OsNAC19* encodes a novel NAC-domain transcription factor and responds to infection by *Magnaporthe grisea*. Plant Sci. 172, 120–130 10.1016/j.plantsci.2006.07.019

[B66] Liu J. L. Wang X. J. Mitchell T. Hu Y. J. Liu X. L. Dai L. Y. (2010). Recent progress and understanding of the molecular mechanisms of the rice *Magnaporthe oryzae* interaction. Mol. Plant Pathol. 11, 419–427 10.1111/j.1364-3703.2009.00607.x 20447289 PMC6640493

[B67] Liu Q. L. Xu K. D. Zhao L. J. Pan Y. Z. Jiang B. B. Zhang H. Q. (2011). Overexpression of a novel chrysanthemum NAC transcription factor gene enhances salt tolerance in tobacco. Biotechnol. Lett. 33, 2073–2082 10.1007/s10529-011-0659-8 21660574

[B68] Liu Y. Schiff M. Dinesh-Kumar S. P. (2002). Virus-induced gene silencing in tomato. Plant J. 31, 777–786 10.1046/j.1365-313X.2002.01394.x 12220268

[B69] Lu M. Ying S. Zhang D. F. Shi Y. S. Song Y. C. Wang T. Y. (2012). A maize stress-responsive NAC transcription factor, ZmSNAC1, confers enhanced tolerance to dehydration in transgenic Arabidopsis. Plant Cell Rep. 31, 1701–1711 10.1007/s00299-012-1284-2 22610487

[B70] Lu P. L. Chen N. Z. An R. Su Z. Qi B. S. Ren F. (2007). A novel drought-inducible gene, ATAF1, encodes a NAC family protein that negatively regulates the expression of stress-responsive genes in Arabidopsis. Plant Mol. Biol. 63, 289–305 10.1007/s11103-006-9089-8 17031511

[B72a] Mao C. Ding W. Wu Y. Yu J. He X. Shou H. (2007). Overexpression of a NAC-domain protein promotes shoot branching in rice. New Phytol. 176, 288–298 10.1111/j.1469-8137.2007.02177.x 17888111

[B71] Mao X. Zhang H. Qian X. Li A. Zhao G. Jing R. (2012). TaNAC2, a NAC-type wheat transcription factor conferring enhanced multiple abiotic stress tolerances in Arabidopsis. J. Exp. Bot. 63, 2933–2946 10.1093/jxb/err462 22330896 PMC3350912

[B72] Mengel K. Kirkby E. A. (2001). Boron, in Principles of Plant Nutrition, 5th Edn, eds Mengel K. Kirkby E.( Dordrecht: Kluwer Academic Publishers), 621–638 10.1007/978-94-010-1009-2_18

[B73] Miller G. Shulaev V. Mittler R. (2008). Reactive oxygen signaling and abiotic stress. Plant Physiol. 133, 481–489 10.1111/j.1399-3054.2008.01090.x 18346071

[B74] Mysore K. S. Crasta O. R. Tuori R. P. Folkerts O. Swirsky P. B. Martin G. B. (2002). Comprehensive transcript profiling of Ptoand Prf mediated host defense responses to infection by *Pseudomonas syringae* pv. tomato. Plant J. 32, 299–315 10.1046/j.1365-313X.2002.01424.x 12410809

[B75] Nakashima K. Ito Y. Yamaguchi-Shinozaki K. (2009). Transcriptional regulatory networks in response to abiotic stresses in Arabidopsis and grasses. Plant Physiol. 149, 88–95 19126699 10.1104/pp.108.129791 PMC2613698

[B76] Nakashima K. Tran L. S. VanNguyen D. Fujita M. Maruyama K. Todaka D. (2007). Functional analysis of a NAC-type transcription factor OsNAC6 involved in abiotic and biotic stress-responsive gene expression in rice. Plant J. 51, 617–630 10.1111/j.1365-313X.2007.03168.x 17587305

[B77] Nilsson L. Müller R. Nielsen T. H. (2010). Dissecting the plant transcriptome and the regulatory responses to phosphate deprivation. Physiol. Plant 139, 129–143 10.1111/j.1399-3054.2010.01356.x 20113436

[B78] Nuruzzaman M. Manimekalai R. Sharoni A. M. Satoh K. Kondoh H. Ooka H. (2010). Genome-wide analysis of NAC transcription factor family in rice. Gene 465, 30–44 10.1016/j.gene.2010.06.008 20600702

[B79] Nuruzzaman M. Sharoni A. M. Satoh K. Kondoh H. Hosaka A. Kikuchi S. (2012a). A genome-wide survey of the NAC transcription factor family in monocots and eudicots, in Introduction to Genetics – DNA Methylation, Histone Modification and Gene Regulation (Hong Kong: iConcept Press), ISBN, 978-14775549-4-4.

[B80] Nuruzzaman M. Sharoni A. M. Satoh K. Moumeni A. Venuprasad R. Serraj R. (2012b). Comprehensive gene expression analysis of the NAC gene family under normal growth conditions, hormone treatment, and drought stress conditions in rice using near-isogenic lines (NILs) generated from crossing Aday Selection (drought tolerant) and IR64. Mol. Genet. Genomics 287, 389–410 22526427 10.1007/s00438-012-0686-8 PMC3336058

[B81] Ochiai K. Shimizu A. Okumoto Y. Fujiwara T. Matoh T. (2011). Suppression of a NAC-like transcription factor gene improves boron-toxicity tolerance in rice. Plant Physiol. 156, 1457–1463 10.1104/pp.110.171470 21543724 PMC3135931

[B82] Oh S. K. Lee S. Yu S. H. Choi D. (2005). Expression of a novel NAC domain-containing transcription factor (CaNAC1) is preferentially associated with incompatible interactions between chili pepper and pathogens. Planta 222, 876–887 10.1007/s00425-005-0030-1 16078072

[B83] Ohnishi T. Sugahara S. Yamada T. Kikuchi K. Yoshiba Y. Hirano H. Y. (2005). OsNAC6, a member of the NAC gene family, is induced by various stresses in rice. Genes Genet. Syst. 80, 135–139 16172526 10.1266/ggs.80.135

[B84] Olsen A. N. Ernst H. A. Lo Leggio L. Skriver K. (2005). NAC transcription factors: structurally distinct, functionally diverse. Trends Plant Sci. 10, 1360–1385 10.1016/j.tplants.2004.12.010 15708345

[B85] Ooka H. Satoh K. Doi K. Nagata T. Otomo Y. Murakami K. (2003). Comprehensive analysis of NAC family genes in *Oryza sativa* and *Arabidopsis thaliana*. DNA Res. 10, 239–247 10.1093/dnares/10.6.239 15029955

[B86] Pacak A. Strozycki P. M. Barciszewska-Pacak M. Alejska M. Lacomme C. Jarmołowski A. (2010). The brome mosaic virus-based recombination vector triggers a limited gene silencing response depending on the orientation of the inserted sequence. Arch. Virol. 155, 169–179 10.1007/s00705-009-0556-9 19937458

[B87] Paul A. Muoki R. C. Singh K. Kumar S. (2012). CsNAM-like protein encodes a nuclear localized protein and responds to varied cues in tea [*Camellia sinensis* (L.) *O*. *Kuntze].* Gene 502, 69–74 10.1016/j.gene.2012.04.017 22543018

[B88] Peng H. Cheng H. Y. Chen C. Yu X. W. Yang J. N. Gao W. R. (2009). A NAC transcription factor gene of chickpea (*Cicer arietinum*), CarNAC3, is involved in drought stress response and various developmental processes. J. Plant Physiol. 166, 1934–1945 10.1016/j.jplph.2009.05.013 19595478

[B89] Puranik S. Bahadur R. P. Srivastava P. S. Prasad M. (2011). Molecular cloning and characterization of a membrane associated NAC family gene, SiNAC from foxtail millet [*Setaria italica* (L.) *P*. *Beauv*]. Mol. Biotechnol. 49, 138–150 10.1007/s12033-011-9385-7 21312005

[B90] Purkayastha A. Dasgupta I. (2009). Virus-induced gene silencing: a versatile tool for discovery of gene functions in plants. Plant Physiol. Biochem. 47, 967–976 10.1016/j.plaphy.2009.09.001 19783452

[B91] Qiu D. Xiao J. Ding X. Xiong M. Cai M. Cao Y. (2007). OsWRKY13 mediates rice disease resistance by regulating defense-related genes in salicylate- and jasmonate-dependent signaling. Mol. Plant Microbe Interact. 20, 492–499 10.1094/MPMI-20-5-0492 17506327

[B92] Ramegowda V. Senthil-Kumar M. Nataraja K. N. Reddy M. K. Mysore K. S. Udayakumar M. (2012). Expression of a finger millet transcription factor, EcNAC1, in tobacco confers abiotic stress-tolerance. PLoS ONE 7:e40397 10.1371/journal.pone.0040397 22808152 PMC3394802

[B93] Ren T. Qu F. Morris T. J. (2000). HRT gene function requires interaction between a NAC protein and viral capsid protein to confer resistance to *turnip crinkle* virus. Plant Cell 12, 1917–1925. 11041886 10.1105/tpc.12.10.1917 PMC149129

[B94] Ren T. Qu F. Morris T. J. (2005). The nuclear localization of the Arabidopsis transcription factor TIP is blocked by its interaction with the coat protein of *Turnip crinkle virus*. Virology 331, 316–324 10.1016/j.virol.2004.10.039 15629774

[B95] Reyes J. C. Muro-Pastor M. I. Florencio F. J. (2004). The GATA family of transcription factors in Arabidopsis and rice. Plant Physiol. 134, 1718–1732 10.1104/pp.103.037788 15084732 PMC419845

[B96] Robertson M. (2004). Two transcription factors are negative regulators of gibberellin response in the HvSPY-signaling pathway in barley aleurone. Plant Physiol. 136, 2747–2761 10.1104/pp.104.041665 15347799 PMC523338

[B97] Rushton P. J. Bokowiec M. T. Han S. Zhang H. Brannock J. F. Chen X. (2008). Tobacco transcription factors: novel insights into transcriptional regulation in the Solanaceae. Plant Physiol. 147, 280–295 10.1104/pp.107.114041 18337489 PMC2330323

[B98a] Saga H. Ogawa T. Kai K. Suzuki H. Ogata Y. Sakurai N. (2012). Identification and characterization of ANAC042, a transcription factor family gene involved in the regulation of camalexin biosynthesis in Arabidopsis. Mol. Plant Microbe. Interact. 25, 684–696 10.1094/MPMI-09-11-0244 22295908

[B98] Satoh K. Shimizu T. Kondoh H. Hiraguri A. Sasaya T. Choi I. R. (2011). Relationship between symptoms and gene expression induced by the infection of three strains of rice dwarf virus. PLoS ONE 3:e18094 10.1371/journal.pone.0018094 21445363 PMC3062569

[B99] Scofield S. R. Nelson R. S. (2009). Resources for virus-induced gene silencing in the grasses. Plant Physiol. 149, 152–157 10.1104/pp.108.128702 19126708 PMC2613721

[B100] Scofield S. R. Huang L. Brandt A. S. Gill B. S. (2005). Development of a virus-induced gene-silencing system for hexaploid wheat and its use in functional analysis of the Lr21-mediated leaf rust resistance pathway. Plant Physiol. 138, 2165–2173 10.1104/pp.105.061861 16024691 PMC1183404

[B101] Selth L. A. Dogra S. C. Rasheed M. S. Healy H. Randles J. W. Rezaian M. A. (2005). A NAC domain protein interacts with *Tomato leaf curl virus* replication accessory protein and enhances viral replication. Plant Cell 17, 311–325 15608335 10.1105/tpc.104.027235 PMC544507

[B102] Seo P. J. Kim M. J. Park J. Y. Kim S. Y. Jeon J. Lee Y. H. (2010). Cold activation of a plasma membrane-tethered NAC transcription factor induces a pathogen resistance response in Arabidopsis. Plant J. 61, 661–671 19947982 10.1111/j.1365-313X.2009.04091.x

[B102a] Seo P. J. Park C. M. (2011). Signaling linkage between environmental stress resistance and leaf senescence in Arabidopsis. Plant Signal Behav. 6, 1564–1566 10.4161/psb.6.10.17003 21921691 PMC3256385

[B103] Shahnejat-Bushehri S. Mueller-Roeber B. Balazadeh S. (2012). Arabidopsis NAC transcription factor JUNGBRUNNEN1 affects thermomemory-associated genes and enhances heat stress tolerance in primed and unprimed conditions. Plant Signal. Behav. 7, 1518–1521 10.4161/psb.22092 23073024 PMC3578882

[B104] Shinozaki K. Yamaguchi-Shinozaki K. Seki M. (2003). Regulatory network of gene expression in the drought and cold stress responses. Curr. Opin. Plant Biol. 6, 410–417 10.1016/S1369-5266(03)00092-X 12972040

[B105] Sindhu A. Chintamanani S. Brandt A. S. Zanis M. Scofield S. R. Johal G. S. (2008). A guardian of grasses: specific origin and conservation of a unique disease-resistance gene in the grass lineage. Proc. Natl. Acad. Sci. U.S.A. 105, 1762–1767 10.1073/pnas.0711406105 18230731 PMC2234218

[B106] Skamnioti P. Gurr S. J. (2009). Against the grain: safeguarding rice from rice blast disease. Trends Biotechnol. 27, 141–150 10.1016/j.tibtech.2008.12.002 19187990

[B107] Souer E. van Houwelingen A. Kloos D. Mol J. Koes R. (1996). The no apical meristem gene of Petunia is required for pattern formation in embryos and flowers and is expressed at meristem and primordia boundaries. Cell 85, 159–170 10.1016/S0092-8674(00)81093-4 8612269

[B108] Sperotto R. A. Ricachenevsky F. K. Duarte G. L. Boff T. Lopes K. L. Sperb E. R. (2009). Identification of up-regulated genes in flag leaves during rice grain filling and characterization of *OsNAC5*, a new ABA-dependent transcription factor. Planta 230, 985–1002 10.1007/s00425-009-1000-9 19697058

[B109] Sun L. Zhang H. Li D. Huang L. Hong Y. Ding X. S. (2013). Functions of rice NAC transcriptional factors, ONAC122 and ONAC131, in defense responses against *Magnaporthe grisea*. Plant Mol. Biol. 81, 41–56 10.1007/s11103-012-9981-3 23103994

[B110] Takasaki H. Maruyama K. Kidokoro S. Ito Y. Fujita Y. Shinozaki K. (2010). The abiotic stress-responsive NAC-type transcription factor OsNAC5 regulates stress-inducible genes and stress tolerance in rice. Mol. Genet. Genomics 284, 173–183 10.1007/s00438-010-0557-0 20632034

[B111] Tang Y. Liu M. Gao S. Zhang Z. Zhao X. Zhao C. (2012). Molecular characterization of novel TaNAC genes in wheat and overexpression of TaNAC2a confers drought tolerance in tobacco. Physiol. Plant 144, 210–224 10.1111/j.1399-3054.2011.01539.x 22082019

[B112] Tian C. Wan P. Sun S. Li J. Che M. (2004). Genome-wide analysis of the GRAS gene family in rice and Arabidopsis. Plant Mol. Biol. 54, 519–532 10.1023/B:PLAN.0000038256.89809.57 15316287

[B113] Tran L. S. Nakashima K. Sakuma Y. Simpson S. D. Fujita Y. Maruyama K. (2004). Isolation and functional analysis of Arabidopsis stress inducible NAC transcription factors that bind to a drought responsive *cis*-element in the early responsive to dehydration stress 1 promoter. Plant Cell 16, 2481–2498 10.1105/tpc.104.022699 15319476 PMC520947

[B114] Uauy C. Distelfeld A. Fahima T. Blechl A. Dubcovsky J. (2006). A *NAC* gene regulating senescence improves grain protein, zinc, and iron content in wheat. Science 314, 1298–1301 10.1126/science.1133649 17124321 PMC4737439

[B115] Udupa S. M. Robertson L. D. Weigand F. Baum M. Kahl G. (1999). Allelic variation at (TAA)n microsatellite loci in a world collection of chickpea (*Cicer arietinum* L.) germplasm. Mol. Gen. Genet. 261, 354–363 10.1007/s004380050976 10102371

[B116] Valent B. Khang C. H. (2010). Recent advances in rice blast effector research. Curr. Opin. Plant Biol. 13, 434–441 10.1016/j.pbi.2010.04.012 20627803

[B117] van der Linde K. Kastner C. Kumlehn J. Kahmann R. Doehlemann G. (2011). Systemic virus-induced gene silencing allows functional characterization of maize genes during biotrophic interaction with Ustilago maydis. New Phytol. 189, 471–483 10.1111/j.1469-8137.2010.03474.x 21039559

[B118] van Loon L. C. Rep M. Pieterse C. M. (2006). Significance of inducible defense-related proteins in infected pants. Annu. Rev. Phytopathol. 44, 135–162 10.1146/annurev.phyto.44.070505.143425 16602946

[B119] Wang X. Basnayake B. M. Zhang H. Li G. Li W. Virk N. (2009a). The Arabidopsis ATAF1, a NAC transcription factor, is a negative regulator of defense responses against necrotrophic fungal and bacterial pathogens. Mol. Plant Microbe Interact. 22, 1227–1238 19737096 10.1094/MPMI-22-10-1227

[B120] Wang X. Goregaoker S. P. Culver J. N. (2009b). Interaction of the *Tobacco mosaic virus* replicase protein with a NAC domain transcription factor is associated with the suppression of systemic host defenses. J. Virol. 83, 9720–9730 19625399 10.1128/JVI.00941-09 PMC2748025

[B121] Waters B. M. Uauy C. Dubcovsky J. Grusak M. A. (2009). Wheat (*Triticum aestivum*) NAM proteins regulate the translocation of iron, zinc, and nitrogen compounds from vegetative tissues to grain. J. Exp. Bot. 60, 4263–4274 19858116 10.1093/jxb/erp257

[B122] Wu Y. Deng Z. Lai J. Zhang Y. Yang C. Yin B. (2009). Dual function of Arabidopsis ATAF1 in abiotic and biotic stress responses. Cell Res. 19, 1279–1290 10.1038/cr.2009.108 19752887

[B123] Xia N. Zhang G. Liu X. Y. Deng L. Cai G. L. Zhang Y. (2010a). Characterization of a novel wheat NAC transcription factor gene involved in defense response against stripe rust pathogen infection and abiotic stresses. Mol. Biol. Rep. 37, 3703–3712 20213512 10.1007/s11033-010-0023-4

[B124] Xia N. Zhang G. Sun Y. F. Zhua L. Xub L. S. Chen X. M. (2010b). TaNAC8, a novel NAC transcription factor gene in wheat, responds to stripe rust pathogen infection and abiotic stresses. Physiol. Mol. Plant Pathol. 74, 394–402 20213512

[B125] Xie Q. Frugis G. Colgan D. Chua N. (2000). Arabidopsis NAC1 transduces auxin signal downstream of TIR1 to promote lateral root development. Genes Dev. 14, 3024–3036 10.1101/gad.852200 11114891 PMC317103

[B126] Xie Q. Sanz-Burgos A. P. Guo H. Garcia J. A. Gutierrez C. (1999). GRAB proteins, novel members of the NAC domain family, isolated by their interaction with a geminivirus protein. Plant Mol. Biol. 39, 647–656 10.1023/A:1006138221874 10350080

[B127] Xue G. P. Way H. M. Richardson T. Drenth J. Joyce P. A. McIntyre C. L. (2011). Overexpression of TaNAC69 leads to enhanced transcript levels of stress up-regulated genes and dehydration tolerance in bread wheat. Mol. Plant 4, 697–712 10.1093/mp/ssr013 21459832

[B128] Yamaguchi M. Kubo M. Fukuda H. Demura T. (2008). Vascularrelated NAC-DOMAIN7 is involved in the differentiation of all types of xylem vessels in Arabidopsis roots and shoots. Plant J. 55, 652–664 10.1111/j.1365-313X.2008.03533.x 18445131

[B129] Yokotani N. Ichikawa T. Kondou Y. Matsui M. Hirochika H. Iwabuchi M. (2009). Tolerance to various environmental stresses conferred by the salt-responsive rice gene ONAC063 in transgenic Arabidopsis. Planta 229, 1065–1075 10.1007/s00425-009-0895-5 19225807

[B130] Yoo S. Y. Kim Y. Kim S. Y. Lee J. S. Ahn J. H. (2007). Control of flowering time and cold response by a NAC-Domain protein in arabidopsis. PLoS ONE 2:e642 10.1371/journal.pone.0000642 17653269 PMC1920552

[B131] Yoon H. K. Kim S. G. Kim S. Y. Park C. M. (2008). Regulation of leaf senescence by NTL9-mediated osmotic stress signaling in Arabidopsis. Mol. Cells 25, 438–445 18443413

[B132] Yoshii M. Shimizu T. Yamazaki M. Higashi T. Miyao A. Hirochika H. (2009). Disruption of a novel gene for a NAC-domain protein in rice confers resistance to rice dwarf virus. Plant J. 57, 615–625 10.1111/j.1365-313X.2008.03712.x 18980655

[B133] Yoshii M. Yamazaki M. Rakwal R. Kishi-Kaboshi M. Miyao A. Hirochika H. (2010). The NAC transcription factor RIM1 of rice is a new regulator of jasmonate signaling. Plant J. 61, 804–815 10.1111/j.1365-313X.2009.04107.x 20015061

[B134] You J. Zong W. Li X. Ning J. Hu H. Li X. (2013). The SNAC1-targeted gene OsSRO1c modulates stomatal closure and oxidative stress tolerance by regulating hydrogen peroxide in rice. J. Exp. Bot. 64, 569–583 10.1093/jxb/ers349 23202132 PMC3542048

[B135] Yuan Y. Zhong S. Li Q. Zhu Z. Lou Y. Wang L. (2007). Functional analysis of rice NPR1-like genes reveals that OsNPR1/NH1 is the rice orthologue conferring disease resistance with enhanced herbivore susceptibility. Plant Biotechnol. J. 5, 313–324 10.1111/j.1467-7652.2007.00243.x 17309686

[B137a] Zhao C. Avci U. Grant E. H. Haigler C. H. Beers E. P. (2008). XND1, a member of the NAC domain family in Arabidopsis thaliana, negatively regulates lignocellulose synthesis and programmed cell death in xylem. Plant J. 53, 425–436 10.1111/j.1365-313X.2007.03350.x 18069942

[B136] Zheng X. Chen B. Lu G. Han B. (2009). Overexpression of a NAC transcription factor enhances rice drought and salt tolerance. Biochem. Biophys. Res. Commun. 379, 985–989 10.1016/j.bbrc.2008.12.163 19135985

[B136a] Zheng X. Y. Spivey N. W. Zeng W. Liu P. P. Fu Z. Q. Klessig D. F. (2012). Coronatine promotes Pseudomonas syringae virulence in plants by activating a signaling cascade that inhibits salicylic acid accumulation. Cell Host. Microbe. 11, 587–596 10.1016/j.chom.2012.04.014 22704619 PMC3404825

[B137] Zhou H. Li S. Deng Z. Wang X. Chen T. Zhang J. (2007). Molecular analysis of three new receptor-like kinase genes from hexaploid wheat and evidence for their participation in the wheat hypersensitive response to stripe rust fungus infection. Plant J. 52, 420–434 10.1111/j.1365-313X.2007.03246.x 17764502

[B138] Zhou M. Li D. Li Z. Hu Q. Yang C. Zhu L. (2013). Constitutive expression of a miR319 gene alters plant development and enhances salt and drought tolerance in transgenic creeping bentgrass. Plant Physiol. 161, 1375–1391 10.1104/pp.112.208702 23292790 PMC3585603

[B139] Zimmermann P. Hirsch-Hoffmann M. Hennig L. Gruissem W. (2004). Genevestigator. Arabidopsis microarray database and analysis toolbox. Plant Physiol. 136, 2621–2632 10.1104/pp.104.046367 15375207 PMC523327

